# Reinfection and complication rates in two-stage revision for chronic hip periprosthetic joint infections using the direct anterior approach

**DOI:** 10.5194/jbji-11-513-2026

**Published:** 2026-08-19

**Authors:** Sebastian Simon, Jennyfer A. Mitterer, Bernhard J. H. Frank, Stephanie Huber, Sujeesh Sebastian, Susana Gardete-Hartmann, Jochen G. Hofstaetter

**Affiliations:** 1 Michael Ogon Laboratory for Orthopedic Research, Orthopedic Hospital Vienna Speising, Vienna, Austria; 2 Center for Anatomy and Cell Biology, Medical University of Vienna, Vienna, Austria; 3 Center for Musculoskeletal Infections, University Hospital Basel, Basel, 4031, Switzerland; 4 Second Department, Orthopedic Hospital Vienna Speising, Vienna, Austria; 5 School of Medicine, Sigmund Freud University Vienna, Freudplatz 1, 1020 Vienna, Austria

## Abstract

**Introduction:** The direct anterior approach (DAA) used in primary total hip arthroplasty (THA) is gaining popularity and has infection rates comparable to other surgical approaches. While two-stage revision remains the gold standard for chronic periprosthetic joint infection (PJI), little data exist on the DAA. This study evaluated reinfection, and complication rates of two-stage septic revisions performed using the DAA.

**Methods:** This retrospective single-center study included data from 8476 patients who underwent primary THA using the DAA between 2013 and 2024. All patients who underwent a two-stage procedure for chronic PJI were included. Clinical outcomes, complication rates, and microbiological spectrum were assessed. All patients received an antibiotic-loaded cement spacer at first stage, followed by antimicrobial therapy.

**Results:** We identified 36 / 8476 (0.4 %) patients (female: 41.7 %, male: 58.3 %) who underwent septic two-stage revision after primary DAA THA. Infection-free survival was 83.4 % at a median 6.2-year follow-up. Successful second-stage reimplantation occurred in 28 / 36 patients (77.8 %), while 2 / 36 (5.6 %) remained infection free after a 1.5-stage procedure due to being clinically unfit for reimplantation. Four patients (11.1 %) underwent a second stage but developed reinfection, requiring septic revision. Two patients (5.6 %) had failed first stage and underwent a Girdlestone procedure. The reinfection rate was 11.1 %, all culture positive at the second stage. Spacer-related complications occurred in 2 / 36 (5.6 %), including one dislocation (2.8 %) and one spacer fracture (2.8 %). Revision for dislocation after the second stage was required in 3 / 36 (8.3 %). Patients with successful reimplantation were younger ( P< 0.001). Most common microorganisms were *Cutibacterium* spp. (28.8 %) and coagulase-negative *Staphylococci* (26.9 %).

**Conclusions:** Two-stage septic revision using the DAA shows reinfection rates comparable to other approaches, with low rates of spacer dislocation and postoperative dislocation after reimplantation.

## 1 Introduction

In recent years, the direct anterior approach (DAA) has become more and more popular in primary total hip arthroplasty (THA), showing good clinical results (Australian Orthopaedic Association National Joint Replacement Registry, 2024; Wilson et al., 2025). Periprosthetic joint infection (PJI) remains one of the most devastating complications after THA, often resulting in increasing patient morbidity and complex management challenges. While the rate of PJI between the DAA and other approaches (Acuña et al., 2021; Chalmers et al., 2023; Wernecke et al., 2024) may not be different, variability in the microbiological spectrum was found (Aichmair et al., 2022; Mitterer et al., 2025). Performing revision THA using the DAA is still under debate, with surgeons preferring other approaches (Australian Orthopaedic Association National Joint Replacement Registry, 2024; Carli et al., 2018; Chalhoub et al., 2025; SIRIS Foundation and ANQ, 2024).

Although one-stage revisions have been gaining popularity in recent years (Goh et al., 2025; Zahar et al., 2019), the two-stage revision continues to be the gold standard for the management of chronic PJIs (Piuzzi et al., 2025; Scuderi et al., 2025). The two-stage approach involves, in the first stage, the removal of all components, thorough surgical debridement, and the implantation of an antibiotic-loaded cement spacer. This is followed by a second stage consisting of repeat debridement and reimplantation of a new arthroplasty once infection control is achieved (Thaler et al., 2020).

The performance of the two-stage procedure in infected THA has been described using different surgical approaches, including the posterior, modified *Hardinge*, and modified *Watson-Jones* methods (Manrique et al., 2014; Neumann et al., 2012; Tsung et al., 2014). The early re-revision rate in two-stage procedures has been reported to be lower than that observed in one-stage revisions (Lenguerrand et al., 2023). Reported success rates for two-stage exchange arthroplasty using antibiotic-loaded spacers range between 70 % and 90 % (Petis et al., 2019; Thakrar et al., 2019).

There is a potential benefit effect of performing a two-stage procedure using the DAA, given its established association with a lower dislocation rate in primary THA compared to other approaches (Charney et al., 2020; Leibovitch et al., 2024). In general, there is little literature available on revision THA using the DAA and even less data are available on the management of PJI using DAA (Kort et al., 2020).

The purpose of this retrospective study was to evaluate the re-revision and the reinfection rate as well as the spacer complication rate in two-stage septic revisions of chronic PJI using the DAA. Moreover, potential difference in the microbiological spectrum were assessed.

## 2 Methods and materials

This retrospective single-center cohort study was approved by the institutional review board (EK11/2020). This study analyzed data from our prospectively maintained institutional arthroplasty registry. We identified a total of 8476 THAs using the DAA between 2013 and 2024.

The standard procedure for managing chronic PJI at our institution is a two-stage revision protocol. This involves the removal of all components, thorough the debridement of infected tissue and implantation of an antibiotic-loaded cement spacer during the first stage. Spacers were used whenever possible, except in patients with poor bone stock or in those with a planned persistent Girdlestone situation. Systemic antibiotic therapy is then administered, based on the microbiological findings and infectious disease specialist's recommendation. Reimplantation is performed once clinical, laboratory, and microbiological parameters indicate infection control.

The indications for a two-stage revision were as follows: the presence of one of two major criteria or a score of 3 to ≥ 6 (3–5: possible infected; ≥ 6: infected) according to the ICM (International Consensus Meeting) 2018 criteria for PJI, in conjunction with a chronic PJI ( > 4 weeks after primary THA) (Shohat et al., 2019).

The inclusion criteria were (1) chronic septic revisions where the primary THA was performed using the DAA (data were pooled for bikini incision and longitudinal incision), (2) patients who had undergone septic two-stage revision using the DAA during both stages, (3) cases with an antibiotic-loaded spacer placed during the first stage. Exclusion criteria included patients who underwent single-stage septic revision, DAIR (debridement, antibiotics, and implant retention) procedures, and those with a history of previous PJI revisions.

During the study period, a total of 52 patients underwent a two-stage revision procedure for chronic PJI at our institution. Of these, 36 (69.2 %) underwent both stages using the DAA, while 13 (25.0 %) were treated using a direct transgluteal approach and 3 (5.8 %) using a posterior approach. The choice of a non-DAA approach was based on individual surgeon preference or previous surgical history.

### 2.1 Clinical workup and microbiology

A hip ultrasound was performed, and, if feasible, an aspiration was carried out preoperatively in accordance with our institutional guidelines, as published elsewhere (Mitterer et al., 2023). Intraoperatively tissue samples (median: 6, range: 4–7) for microbiological and histological analysis were obtained. Samples were sent to microbiological and histopathological analysis, and the explanted implant components were sent for sonication. Postoperatively, ICM 2018 criteria were calculated with all available diagnostic parameters.

Pathogens were classified into two categories: those likely to be contaminants and those not likely to be contaminants (Sousa et al., 2023). The following microorganisms were not likely to be contaminants: *Staphylococcus aureus, Staphylococcus lugdunensis*, Beta-haemolytic Streptococci,* Streptococcus anginosus*,group* Enterococci *spp.*, *Enterobacteriaceae,* Pseudomonas aeruginosa*, anaerobic Gram-negative rods, and *Candida *spp. The following microorganisms were likely to be contaminants: most coagulase-negative Staphylococci spp. (*S. epidermidis, S. capitis* or *S. haemolyticus*), anaerobic Gram-positive bacilli (*Cutibacterium acnes/avidum*), or anaerobic Gram-positive cocci (*Finegoldia magna*).

All patients received empiric routine intravenous (IV) second-generation cephalosporin (or vancomycin for those with a history of allergy to penicillin or cephalosporins) prior to surgical incision as antibiotic prophylaxis.

### 2.2 Spacer implantation (first stage)

Patients received either a custom-made antibiotic-loaded spacer with a cemented stem (Gentamicin + Vancomycin; COPAL, Hereus Medical, Wehrheim, Germany) or a cemented polyethylene (PE) Müller II cup (Zimmer Biomet, Warsaw, IN, USA), or a PE dual mobility head or a pre-formed antibiotic-loaded spacer (Biomed One Stage Spacer, Zimmer Biomet Warsaw, IN, USA), based on surgeons' preference. In medically unfit patients, these custom-made spacers involves the placement of an articulating antibiotic-loaded permanent prosthesis that could remain unless clinical failure necessitates revision (1.5 stage) (Scuderi et al., 2025).

### 2.3 Postoperative protocol and second stage

Following the first and second stage, each postoperative empirical antibiotic treatment, including 2 weeks IV and 4 weeks per os, was applied. This resulted in a minimum duration of 12 weeks antibiotic treatment (Li et al., 2020). Once microbiological test results were available, the regimen was changed according to the recommendations of our infectious disease specialist.

The patients were allowed to bear weight as tolerated without pain. Patients were discharged on oral antibiotics 2 weeks after the first stage of the two-stage procedure, with subsequent C-reactive protein (CRP) measurements taken at 2-week intervals. The second stage was performed after a period of 6 weeks following the first stage. The decision regarding second stage was made in accordance with the infectious disease specialist based on the infecting organism, local wound status, and CRP values (normalized or continuously decreasing).

### 2.4 Follow-up

The infection-free survivorship and the re-revision rate were evaluated. Follow-up was conducted by telephone recall, review of our clinical databases for clinical visits, and review of the Austrian electronic health record (ELGA), including all medical records if revisions were performed elsewhere. The median follow-up was 6.2 (interquartile range (IQR) 3.8–8.1) years. Infection-free success was defined as the absence of clinical signs of infection, no subsequent septic revision surgery, and no requirement for suppressive antibiotic therapy at the latest follow-up.

### 2.5 Statistical analysis

Descriptive statistics were used with the means (M), standard deviation (SD), and median (Md) for continuous study parameters, and frequencies and percentages for categorical variables. When the data were skewed, the IQRs were used. Continuous data were compared using Mann–Whitney* U*-tests or two-sample t -tests for non-parametric and parametric data, respectively. Categorical data were compared using Pearson's chi-square tests or Fisher's exact tests, as appropriate. Statistical significance was two tailed and set at a P value of ≤ 0.05. All analyses were performed using IBM Statistical Package for the Social Sciences (SPSS^®^) Version 25 (Armonk, New York) and GraphPad Prism 8 (GraphPad Software, Boston, Massachusetts).

## 3 Results

**Figure 1:**
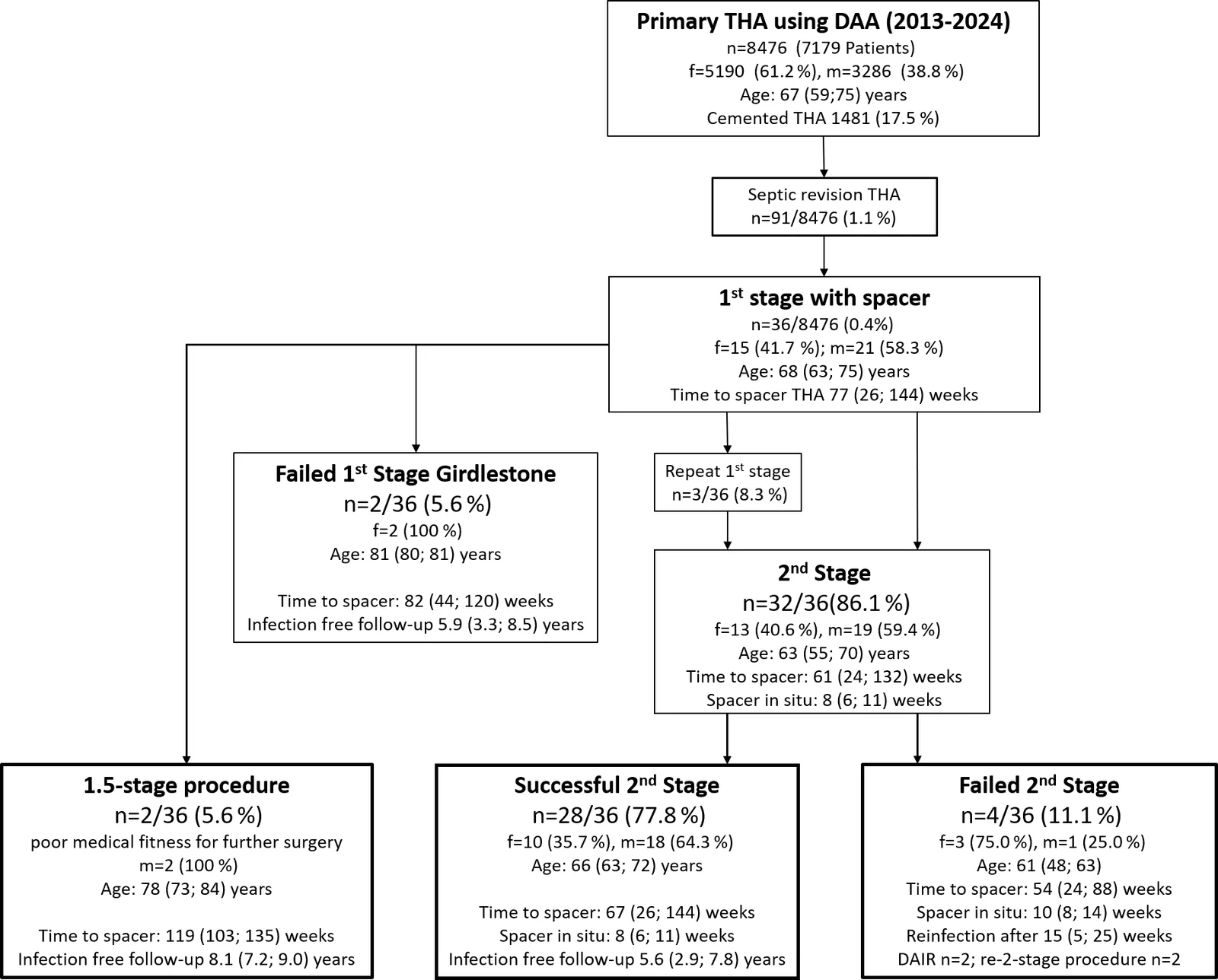
Flow chart for patients after primary total hip arthroplasty (THA) using the direct anterior approach (DAA) and underwent second-stage procedure; f: female, m: male, DAIR: debridement, antibiotics and implant retention. The age is given as the median plus the interquartile range.

In total, 36 out of 8476 (0.4 %) DAA-THA received a first-stage revision with a spacer and were included in the final analysis (Fig. 1). A total of 28 / 36 (77.8 %) patients underwent a successful second stage with reimplantation, and 4 / 36 (11.1 %) patients had a reinfection and required subsequent septic revision after second stage (two DAIR and two re-second-stage procedures). However, a spacer exchange was required before the second-stage procedure in 3 / 36 (8.3 %) patients. In 2 / 36 (5.6 %) patients, a 1.5-stage procedure was performed as definitive treatment due to poor medical fitness for further surgery. Furthermore, 2 / 36 (5.6 %) patients with persistent infections underwent a Girdlestone procedure as definitive treatment (see Table 1). Of the 36 patients, 32 (88.9 %) underwent second-stage reimplantation. At the latest follow-up, 28 / 32 (87.5 %) remained infection free, whereas 4 / 32 (12.5 %) developed reinfection requiring further septic revision surgery (see Table 1).

**Figure 2:**
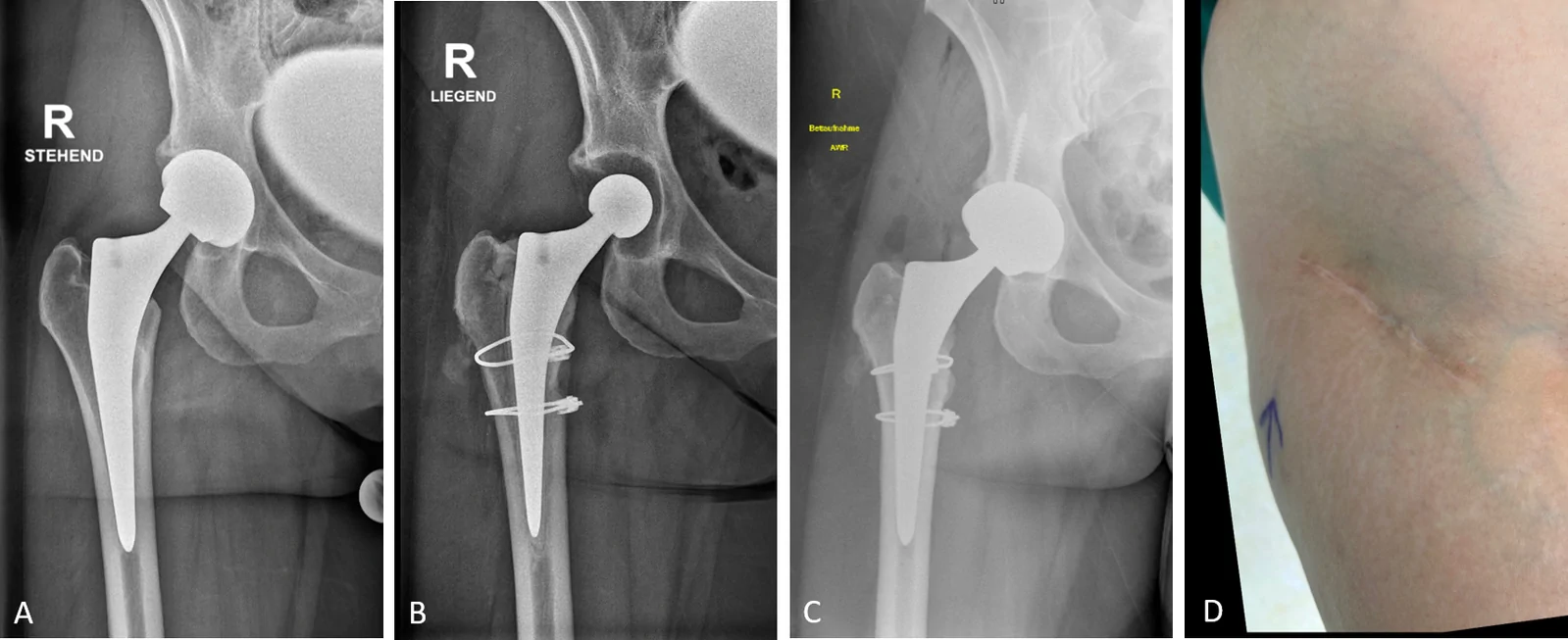
**(A)** shows a postoperative anteroposterior (AP) X-ray of a primary total hip arthroplasty (THA) using the direct anterior approach (DAA) with a bikini incision, **(B)** shows a postoperative ap radiograph with a custom-made spacer and two cable wires after femoral component removal and femoral osteotomy using the DAA, **(C)** shows a postoperative ap radiograph with a successful second-stage reimplantation. **(D)** Scar after 3 × bikini incision DAAs.

**Table 1:** Patient demographics, re-revision rate and complication rate in patients who underwent a second-stage procedure with a spacer as first stage. Mean with SD (standard deviation) and median with IQR (interquartile range). BMI: body mass index. ASA: American Society of Anesthesiologists (1–5).

		Data	n=36
Demographic data		Sex male (%)	21 (58.3 %)
		female (%)	15 (41.7 %)
		Age primary THA (years; median)	66 (60; 75)
		Age first stage (years, median)	68 (63; 75)
		Time to spacer (weeks, median)	77 (26; 144)
		BMI (kg m^−2^)	26.9 ( ± 5.0)
		ASA I (%)	11 (30.6 %)
		II (%)	19 (52.8 %)
		III (%)	6 (16.7 %)
			
Spacer data & Spacer		Duration of spacer (weeks, median)	8 (6; 11)
	complications	Pre-formed spacer (%)	11 (30.6 %)
		Custom-made spacer (%)	25 (69.4 %)
		Femoral osteotomy for stem removal (%)	7 (19.4 %)
		Intraop. femoral fracture at first stage (%)	1 (2.8 %)
		Spacer fracture (%)	1 (2.8 %)
		Spacer dislocation (%)	1 (2.8 %)
		
Outcome		Successful second stage (%)	28 (77.8 %)
		1.5 stage (%)	2 (5.6 %)
		Septic revision after second stage (%)	4 (11.1 %)
		Failed first stage (%)	2 (5.6 %)
		Aseptic revision rate (%)	4 / 36 (11.1 %)
		Revision due to dislocation (%)	3 (8.3 %)
		Postop. periprosthetic femoral fracture (%)	1 (2.8 %)
		Deceased ≤ 5 years post second stage (%)	3 (8.3 %)
		Deceased ≥ 5 years post second stage (%)	2 (5.6 %)

In total, a preformed spacer was used in 11 / 36 (30.6 %) and a custom-made spacer in 25 / 36 (69.4 %) during the first stage of a second-stage procedure. In the preformed spacer group, there was one spacer fracture and one spacer dislocation (2 / 11; 18.2 %). Both patients underwent a spacer exchange. No spacer-related complications occurred in the custom-made spacer group.

During the first stage in 7 / 36 (19.4 %), a femoral osteotomy was necessary for the removal of the femoral component. An additional transgluteal incision was used to perform this osteotomy in two of these cases. In 1 / 36 (2.8 %) patients, intraoperative fracture occurred on the calcar side. For femoral osteotomy and intraoperative fractures, cable wires (Cable-Ready, Zimmer Biomet, Warsaw, IN, USA) were used for secure fixation using the DAA. There was one postoperative fracture 8 d after reimplantation, which was also treated with cable wires.

A total of four patients (4 / 36, 11.1 %) experienced a dislocation after the second-stage procedure. Three of these patients (8.3 %) required revision with head and liner exchange for dislocation at a median of 22 (IQR 13; 47) d. One patient (2.8 %) had a dislocation and was successfully treated with closed reduction (see Table 1).

Patients who underwent successful second-stage reimplantation and remained infection-free at a median follow-up of 6.2 (IQR 3.8; 8.1) were significantly younger (66 IQR 63; 72 years) than those who did not undergo reimplantation (81 IQR 77–84 years; failed fist-stage Girdlestone + 1.5-stage procedures); P< 0.001. No additional confounding factors for failure were identified.

### Microbiology results

In total, 34 out of 36 (94.4 %) first stages were culture positive, and two (5.6 %) were culture negative. A single culture-positive result was observed in 24 / 36 (66.7 %) patients, while a multiple culture-positive result was observed in 12 / 36 (33.3 %) patients. Overall, 30 / 36 (83.3 %) had an ICM score of ≥ 6, and 6 / 36 (16.7 %) had a score between 3 and 5. A total of 52 microorganisms were detected during the first stages with a spacer (see Table 2). The most prevalent microorganisms were *Cutibacterium* spp. (15 / 52; 28.8 %), CoNS (coagulase-negative *Staphylococci*; 16 / 52; 26.9 %), and Gram-negative organisms (12 / 52; 23.1 %). There was no significant difference in the distribution of high- or low-virulence microorganisms between the reimplantation and no-reimplantation (failed first-stage Girdlestone + 1.5-stage procedures) groups ( P> 0.99).

**Table 2:** Microbiological spectrum for patients with successful second stage and those with no reimplantation, ICM 2018: International Consensus Meeting, spp.: species, MRSA: methicillin-resistant *Staphylococcus aureus*. ^∗^ Not likely to be a contaminant.

Parameter	Reimplantation n=31	No reimplantation n=5
Culture positive	29 (93.5 %)	5 (100 %)
Culture negative	2 (6.5 %)	0
Single culture positive	19 (61.3 %)	5 (100 %)
Multiple cultures positive	12 (38.7 %)	0
ICM 2018 infected	25 (80.6 %)	5 (100 %)
ICM 2018 possibly infected	6 (19.4 %)	0
ICM 2018 not infected	0	0
High virulent microorganism PJI	14 (45.2 %)	2 (40.0 %)
Low virulent microorganism PJI	17 (54.8 %)	3 (60.0 %)
No. of isolated microorganisms	47	5
Gram-positive bacteria	36 (76.6 %)	4 (80.0 %)
*Staphylococcus epidermidis*	9	2
*Cutibacterium acnes*	6	–
*Cutibacterium avidum*	9	–
*Staphylococcus aureus* ^∗^	4	–
MRSA*	1	–
*Staphylococcus lugdunensis* ^∗^	2	–
*Staphylococcus capitis*	–	1
*Alpha haemolytic streptococci* ^∗^	2	–
*Enterococcus faecalis* ^∗^	1	1
*Bacillus* spp.	2	–
Gram-negative bacteria	11 (23.4 %)	1 (20.0 %)
*Pseudomonas aeruginosa* ^∗^	1	–
*Finegoldia magna* ^∗^	1	–
*Moraxella osloensis*	1	–
*Proteus mirabilis* ^∗^	2	1
*Citrobacter koseri* ^∗^	1	–
*Escherichia coli* ^∗^	1	–
*Enterobacter cloacae* ^∗^	2	–
*Klebsiella pneumoniae* ^∗^	1	–
*Morganella morganii* ^∗^	1	–

Of the 28 out of 36 patients (77.8 %) with a successful second stage, 5 / 28 (17.9 %) had a culture-positive second stage. An additional 5 / 28 (17.9 %) patients had a culture-positive second stage, with CoNS or *Cutibacterium* spp. after enrichment. None required a septic re-revision during follow-up. An example of a patient following two-stage revision is shown in Fig. 2.

All patients who underwent septic re-revision had a culture-positive second stage (4 / 4; 100 %). Interestingly, of the 14 culture-positive second-stage cases, 9 (64.3 %) had different microorganisms, 2 / 14 (14.3 %) had additional microorganisms, and 3 / 14 (21.4 %) had the same microorganisms as those found during the first stage.

## 4 Discussion

In this study, we evaluated patients who underwent a two-stage procedure using a DAA for the treatment of chronic PJI following DAA THR.

Of the 32 patients who underwent second-stage reimplantation, 28 (87.5 %) remained infection free at the latest follow-up, while 4 patients (12.5 %) developed reinfection requiring further septic revision surgery. These results are consistent with reinfection rates in two-stage procedures from other studies, which range from 8 % to 32 % (Corona et al., 2020; Goumenos et al., 2024; Hartman et al., 2022; Kandel et al., 2019; Petis et al., 2019; Triantafyllopoulos et al., 2017). However, most of these studies do not specify the approach or provide detailed information on it. As the DAA becomes more common, more revisions will be performed through the DAA. This study shows no higher reinfection rate when performing a two-stage procedure in chronic PJI patients using the DAA. In our study, all two-stage procedures were performed after primary DAA THA. The DAA may be safely reused when considered appropriate by the treating surgeon (Manrique et al., 2014). The approach discordance between primary and revision THA is more frequent when the DAA is used for primary THA (Harmer et al., 2022). The DAA can be valuable in preserving muscular tissues that have already been compromised by previous surgeries or the infection itself.

Implant instability is another concern after two-stage procedures. In this study, 8.3 % of patients required a revision due to dislocation after reimplantation. This is slightly lower compared to the dislocation rate in other studies after two-stage procedures, described between 9 % and 30 % (Abuelnour et al., 2025; Finucane et al., 2020; McAlister et al., 2019). However, these studies described two-stage revisions that utilize the direct lateral or posterior approach. In the study by Thaler et al., a DAA was utilized for a two-stage procedure, and the dislocation rate was determined to be zero. Nevertheless, the study revealed that 12.2 % of patients experienced spacer dislocation (Thaler et al., 2020). The review of mechanical complications of hip spacers found that dislocation occurred in 10.8 % of cases (Sambri et al., 2023). One patient (2.6 %) in this study had a spacer dislocation. The low spacer dislocation rate and the low dislocation rate after reimplantation in this study may be partially explained by the DAA.

In this study, only one femoral fracture (2.8 %) occurred during explantation at the calcar region, and one patient suffered a postoperative periprosthetic fracture (2.6 %). The fracture rate in our cohort was lower than that reported in previous studies, which ranged from 10 % and 15 % (Jaubert et al., 2022; Petis et al., 2019; Thaler et al., 2020). This could be explained by the high number of femoral osteotomies (19.4 %) performed for explantation of the stem in this study, which resulted in a low fracture risk even when using the DAA. In addition, all fracture occurrences within our cohort have been treated via the DAA.

In addition, the incidence of spacer fracture in this study was only 2.8 %, which is significantly lower than the 8 % rate documented in the literature (Jaubert et al., 2022; Jones et al., 2019). One potential explanation for this finding is the relatively high incidence of custom-made spacers in this study. In the study conducted by Thaler et al., only custom-made spacers were used, and they documented no-spacer fracture (Thaler et al., 2020). In total, spacer-related complications were low in this study. Consequently, the use of a custom-made spacer could prevent spacer-related complications.

In comparison with other studies, this study showed low dislocation, low reinfection, and low spacer complication rates. These findings suggest that the DAA is not inferior to other approaches in two-stage revision surgery.

There are several limitations to this study that should be acknowledged. The retrospective design inherently carries the risk of selection bias. Additionally, the relatively low sample size limits the statistical power of our findings, particularly in subgroup analyses such as spacer type or microbiological spectrum. The inclusion of a heterogeneous patient cohort may further influence the interpretation of outcomes. Nevertheless, this study provides 5-year follow-up data using a standardized preoperative and postoperative protocol, which strengthens the reliability of conclusions regarding recurrence and complication rates.

## 5 Conclusions

In this study, the reinfection rate following two-stage revision using the DAA was low, underscoring its effectiveness in managing chronic PJIs after primary DAA THA. In addition, the DAA was associated with a low incidence of other complications, including spacer-related issues, fractures, and dislocations.

## Data Availability

The datasets generated and analyzed during the current study are not publicly available due to institutional and ethical data protection regulations but are available from the corresponding author on reasonable request, subject to approval by the local ethics committee and institutional data governance policies. The statistical code used for data analysis is available from the corresponding author on reasonable request.
